# Measurement properties of quality of life measurement instruments for infants, children and adolescents with eczema: protocol for a systematic review

**DOI:** 10.1186/s13643-016-0202-z

**Published:** 2016-02-09

**Authors:** Daniel Heinl, Cecilia A. C. Prinsen, Aaron M. Drucker, Robert Ofenloch, Rosemary Humphreys, Tracey Sach, Carsten Flohr, Christian Apfelbacher

**Affiliations:** Medical Sociology, Department of Epidemiology and Preventive Medicine, University of Regensburg, Regensburg, Germany; Department of Epidemiology and Biostatistics, EMGO+ Institute for Health and Care Research, VU University Medical Center, Amsterdam, The Netherlands; Department of Dermatology, The Warren Alpert Medical School of Brown University, Providence, RI USA; Department of Clinical Social Medicine, University Hospital Heidelberg, Heidelberg, Germany; Budleigh Salterton, Devon UK; Norwich Medical School, University of East Anglia, Norwich, UK; St John’s Institute of Dermatology, King’s College London, London, UK; Division of Public Health and Primary Care, Brighton and Sussex Medical School, Falmer, UK

**Keywords:** Eczema, Atopic dermatitis, Measurement instruments, Health-related quality of life, Quality of life, Validity, Reliability, Responsiveness, Interpretability

## Abstract

**Background:**

Eczema is a common chronic or chronically relapsing, inflammatory skin disease that exerts a substantial negative impact on quality of life (QoL). The Harmonising Outcome Measures for Eczema (HOME) initiative has used a consensus-based process which identified QoL as one of the four core outcome domains to be assessed in all eczema clinical trials. A number of measurement instruments exist to measure QoL in infants, children, and adolescents with eczema, and there is a great variability in both content and quality of the instruments used. Therefore, the objective of the proposed research is to comprehensively and systematically assess the measurement properties of the existing measurement instruments that were developed and/or validated for the measurement of patient-reported QoL in infants, children, and adolescents with eczema.

**Methods/design:**

This study is a systematic review of the measurement properties of patient-reported measures of QoL developed and/or validated for infants, children, and adolescents with eczema. A systematic literature search will be carried out in MEDLINE via PubMed and EMBASE using a selection of relevant search terms. Eligible studies will be primary empirical studies evaluating, describing, or comparing measurement properties of QoL instruments for infants, children, and adolescents with eczema. Two reviewers will independently perform eligibility assessment and data abstraction. Evidence tables will be used to record study characteristics, instrument characteristics, measurement properties, and interpretability. The adequacy of the measurement properties will be assessed using predefined criteria. The COnsensus-based Standards for the selection of health Measurement INstruments (COSMIN) checklist will be used to evaluate the methodological quality of included studies. A best evidence synthesis will be undertaken if more than one study has examined a particular measurement property.

**Discussion:**

The proposed systematic review will yield a comprehensive assessment of measurement properties of existing QoL instruments in infants, children, and adolescents with eczema. The results will serve as a basis to recommend a QoL measurement instrument for infants, one for children, and one for adolescents for use in future clinical trials.

**Systematic review registration:**

PROSPERO CRD42015023483

**Electronic supplementary material:**

The online version of this article (doi:10.1186/s13643-016-0202-z) contains supplementary material, which is available to authorized users.

## Background

Eczema (synonymous with atopic eczema, atopic dermatitis) represents the most common chronic disease in children in many countries [1]. Its main symptom is persistent pruritus [2]. The disease has a negative impact on the quality of life (QoL) of the patients and their families [3, 4]. Despite the fact that various interventions exist for eczema, uncertainties concerning the best treatment options remain. A major reason for this situation is the inconsistent use of varying eczema outcome measures in randomized controlled trials, making the comparison of interventions across these trials in systematic reviews and meta-analyses difficult. Thus, outcome measures in clinical trials of (pediatric) eczema patients need to be improved [5].

An internationally acknowledged way to ameliorate this unsatisfying situation is the development of a core outcome set (COS) [6]. The Harmonising Outcome Measures for Eczema (HOME) initiative [7] aims to develop a COS for eczema. Clinical signs measured by means of a physician-assessed instrument, symptoms, long-term control of eczema flares, and QoL were agreed on as the core outcome domains to be assessed in all future eczema trials [8, 9]. There was broad international consensus among clinicians, patients, and methodologists that the Outcome Measures in Rheumatology (OMERACT) quality criteria “truth, discrimination, and feasibility” [10] need to be met for eczema outcome measures to be recommended by the HOME initiative [9]. The next crucial step in the process of standardizing eczema outcome measurements is now the identification of appropriate instruments to measure each of the four core outcome domains of atopic eczema [11]. For adult QoL measurement instruments, this has already been undertaken, using methods similar to this proposed review [12]. The results have been published [13]. As the methodology of this systematic review will be in large parts identical to the one applied in the review on adult QoL instruments, content and wording of this protocol are very similar to the published protocol of the review on adult QoL instruments [12]. This pertains specifically to the methods section. To ensure transparency, differences in the methodology of both reviews are highlighted in the “Differences between this review and previously suggested methodology” section.

### Objectives

1. To systematically assess the measurement properties of patient- or parent-reported measurement instruments of QoL for infants, children, and adolescents with eczema
2. To identify outcome measurement instruments for QoL in infants, children, and adolescents with eczema
  - That meet the predefined criteria to be recommended [10, 9] for the measurement of QoL in future eczema trials
  - That have the potential to be recommended in the future depending on the results of further validation studies
  - That do not meet the predefined criteria to be recommended [10, 9] and therefore should not be used any more
3. To provide the evidence base for an international consensus process
  - To further standardize the assessment of QoL in infants, children, and adolescents with eczema in clinical trials
  - To prioritize further research concerning QoL assessment in infants, children, and adolescents with eczema

## Methods/design

### Protocol and registration

The methods for this systematic review have been developed according to the recommendations from the Preferred Reporting Items for Systematic Reviews and Meta-Analyses Protocols (PRISMA-P) statement [14], and a populated PRISMA-P checklist is available as an Additional file 1 to this protocol. This protocol has been registered in the International Prospective Register of Systematic Reviews (PROSPERO): CRD42015023483.

### Literature search

A systematic literature search will be performed in PubMed and EMBASE. The search strategy will contain blocks of search terms related to the following aspects:

1. Construct of interest: quality of life
2. Target population: (atopic) eczema (Table 1)
3. Measurement properties: the precise PubMed search filter for finding studies on measurement properties developed by Terwee et al. will be used to identify relevant articles [15]. This filter has a sensitivity of 93.1 % and a precision of 9.4 %
4. Interpretability

**Table 1:** Inclusion and exclusion criteria

	Inclusion criteria	Exclusion criteria
Population	Eczema (synonyms: atopic eczema, atopic dermatitis, neurodermatitis); populations younger than 16 years of age	Populations with other skin diseases than eczema, populations of adults with eczema, carers of infants/children with eczema
Study design	Development study, validation study	Linguistic validation studies
Outcome	Quality of life, health-related quality of life	Signs, disease severity measure, disease control measure, biomarker, physiology of the skin
Type of measurement instrument	Self- or proxy-reported measurement instrument	All others
Publication type	Articles with available full text	Abstracts

The search will not be restricted with respect to the publication time of retrieved studies. The entire search strategy is available as an Additional file 2 to this protocol. The systematic electronic search will be supplemented by hand searching of reference lists of studies included and key articles on this topic. Furthermore, an additional search will be performed in each database, including the names of the instruments which are found in the initial search. The PROQOLID (www.proqolid.org) database, an online database of QoL instruments, will be searched. The initial search in PubMed and EMBASE will be carried out on a single day that will be reported in the final review, whereas the hand searching process will be performed during the eligibility assessment of articles, which may take several weeks. The additional search of each database will be done, after the eligibility assessment has been completed, on a single day.

### Eligible studies

A study will be included if it is published as a full-text paper and concerns the development (“development paper”) and/or evaluation of the measurement properties (“validation paper”) of instruments that measure QoL or health-related quality of life (HrQoL) in infants, children, and adolescents with eczema. Measurement instruments that assess both the QoL of children and caregivers will be included if separate scores for the QoL of the child and for the QoL of the caregiver can be calculated. Generic QoL measurement instruments for infants, children, and adolescents and measurement instruments assessing solely the QoL of caregivers will not be considered eligible. The HOME initiative decided in 2011 that generic QoL measurement instruments are not eligible for the COS [16]. QoL measurement instruments for caregivers will be investigated in a separate review. To be eligible, at least 50 % of a study’s population must consist of eczema patients younger than 16 years of age. A study with a mixed patient sample will be eligible either if it presents a subgroup analysis for infants, children, and adolescents with eczema or if infants, children, and adolescents with eczema constitute at least 50 % of the study population. The measurement instrument must be a self- or proxy-reported questionnaire. Articles that report indirect evidence, for instance, by using data obtained within the context of a clinical trial, will not be considered eligible. Articles assessing the measurement properties of dermatology-specific instruments in non-eczema samples will not be considered eligible.

### Study selection

Two reviewers will independently judge titles and abstracts retrieved in the literature search and, at a second stage, full-text articles for eligibility (Table 1). Disagreements will be resolved by consensus-seeking discussions within the research team.

### Data extraction

Relevant data from all included articles will be summarized in evidence tables. The evidence tables drafted for the adults’ review [12] will be slightly adapted. Data from each article included will be extracted independently by two reviewers. Reviewers will work in pairs on defined sets of articles. Disagreements will be resolved by consensus-seeking discussions within the research team.

Evidence tables will include the following: reference, geographical location, language, setting, study type, key characteristics of study subjects, name of measurement instruments, domains measured, number of items and (sub)scales, number and type of response categories, recall period in the questions, scoring algorithm, time needed for administration, mode of administration, target population for whom the questionnaire was originally developed, how a full copy of the questionnaire can be obtained, the instructions given to those who complete the questionnaire, the available versions and translations of the questionnaire, results of the measurement properties, all items from the COnsensus-based Standards for the selection of health Measurement INstruments (COSMIN) box Generalisability, and all items from the COSMIN box Interpretability [17, 18].

If general characteristics of an instrument (that is, name of measurement instrument, number of items and (sub)scales, number and type of response categories, recall period in the questions, scoring algorithm, time needed for administration, mode of administration, target population for whom the questionnaire was originally developed, how a full copy of the questionnaire can be obtained, the instructions given to those who complete the questionnaire, the available versions and translations of the questionnaire) cannot be extracted from the studies included, the original development paper may be consulted to obtain missing information.

### Content comparison

An overview of the content of each instrument on content domain level will be presented in order to visualize the content covered by the different instruments. The original development paper will be consulted to obtain this information.

### Assessment of the methodological quality of included studies

The COSMIN checklist [17–19] will be used to evaluate the methodological quality of included studies. In the COSMIN checklist (www.cosmin.nl), four domains are distinguished (reliability, validity, responsiveness, and interpretability) with related measurement properties and aspects of measurement properties. These are listed in Table 2 (adapted from Mokkink LB et al. [18]).

**Table 2:** Definitions of domains, measurement properties, and aspects of measurement properties

Domain	Measurement property	Aspect of a measurement property	Definition
Reliability			The degree to which the measurement is free from measurement error.
Reliability (extended definition)			The extent to which scores for patients who have not changed is the same for repeated measurement under several conditions: for example, using different sets of items from the same HR-PROs (internal consistency), over time (test-retest) by different persons on the same occasion (inter-rater) or by the same persons (i.e., raters or responders) on different occasions (intra-rater).
	Internal consistency		The degree of interrelatedness among the items.
	Reliability		The proportion of total variance in the measurements which is because of “true”^a^ differences among patients.
	Measurement error		The systematic and random error of a patient’s score that is not attributed to true change of the construct to be measured.
Validity			The degree to which an HR-PRO instrument measures the construct(s) it purports to measure.
	Content validity		The degree to which the content of an HR-PRO instrument is an adequate reflection of the construct to be measured.
		Face validity	The degree to which (the items of) an HR-PRO instrument indeed looks as though they are an adequate reflection of the construct to be measured.
	Construct validity		The degree to which the scores of an HR-PRO instrument are consistent with hypotheses (for instance with regard to internal relationships, relationships to scores of other instruments, or differences between relevant groups) based on the assumption that the HR-PRO instrument validly measures the construct to be measured.
		Structural validity	The degree to which the scores of an HR-PRO instrument are an adequate reflection of the dimensionality of the construct to be measured.
		Hypothesis testing	Idem construct validity.
		Cross-cultural validity	The degree to which the performance of the items on a translated or culturally adapted HR-PRO instrument are an adequate reflection of the performance of the items of the original version of the HR-PRO instrument.
Responsiveness			The ability of an HR-PRO instrument to detect change over time in the construct to be measured.
	Responsiveness		Idem responsiveness.
Interpretability^b^			The degree to which one can assign qualitative meaning—that is, clinical or commonly understood connotations—to an instrument’s quantitative scores or changes in scores.

*HR-PROs* health-related patient-reported outcomes, *CTT* classical test theory

^a^The word “true” must be seen in the context of the CTT, which states that any observation is composed of two components—a true score and error associated with the observation. “True” is the average score that would be obtained if the scale were given an infinite number of times. It refers only to the consistency of the score and not to its accuracy [26]

^b^Interpretability is not considered a measurement property but an important characteristic of a measurement instrument

For each measurement property, the COSMIN checklist consists of 5 to 18 items covering methodological standards (organized in nine boxes for the nine measurement properties). In addition, each item can be scored on a 4-point rating scale (that is, “poor,” “fair,” “good,” “excellent”). Taking the lowest rating for each item in one box, an overall quality score (“poor,” “fair,” “good,” “excellent”) is obtained for each measurement property separately [20].

### Assessment of measurement properties and further characteristics of QoL instruments

We will assess all measurement properties from the COSMIN checklist in this review, with the exception of the measurement property “criterion validity,” which will not be considered for the purpose of this systematic review, since there is no gold standard for QoL. Data on interpretability and feasibility will be collected where presented. With the exception of content comparison and instrument characteristics, we will regard different language versions of the same questionnaire separately throughout the review. Our principal reason for doing so is the fact that it is problematic to assume that different language versions of measurement instruments exhibit the same measurement properties. Strictly speaking, it is the measurements themselves that are valid, reliable, and responsive and not the instruments per se*.*

### Assessment of the adequacy of the measurement instruments

The predefined criteria for rating the adequacy of the measurement instruments recommended by the COSMIN group will be used in a slightly modified version [21] (Table 3). These criteria are in accordance with the OMERACT filter [10], which has been adopted by the HOME initiative [9] and applied in a previous review on atopic eczema outcome measures [22]. The measurement property “hypothesis testing” will be split into the aspects convergent/divergent (defined as the correlation between instruments measuring similar/different constructs [23]) and discriminative validity (defined as the ability of a measurement instrument to distinguish between different subgroups of patients [23]) for this review. An overall rating for hypothesis testing will be obtained from both aspects in the end (see “Generating recommendations for the use of QoL measurement instruments for eczema” section). Where studies apply item response theory (IRT) methods in the evaluation of measurement properties, rather than in the development of measurement instruments, we will be able to assess the adequacy and methodological quality of internal consistency, construct validity, structural validity, and cross-cultural validity.

**Table 3:** Adequacy criteria for measurement properties adapted from [21] and [27]

Property	Rating	Adequacy criteria
Reliability		
Internal consistency (CTT methods applied)	+	Cronbach’s alpha(s) ≥0.70
	?	Cronbach’s alpha not determined
	−	Cronbach’s alpha(s) <0.70
Internal consistency (IRT methods applied)	+	Person Separation Index ≥0.70
	?	Person Separation Index not determined
	−	Person Separation Index <0.70
Measurement error	+	MIC > SDC OR MIC outside the LoA
	?	MIC not defined
	−	MIC ≤ SDC OR MIC equals or inside LoA
Reliability	+	ICC/weighted Kappa ≥0.70, OR Pearson’s *r* ≥ 0.80
	?	Neither ICC/weighted Kappa, nor Pearson’s *r* determined
	−	ICC/weighted Kappa <0.70 OR Pearson’s *r* < 0.80
Validity		
Content validity	+	All items are considered to be relevant for the construct to be measured, for the target population, and for the purpose of the measurement AND the questionnaire is considered to be comprehensive
	?	Not enough information available
	−	Not all items are considered to be relevant for the construct to be measured, for the target population, and for the purpose of the measurement OR the questionnaire is considered not to be comprehensive
Construct validity		
Structural validity (CTT methods applied)	+	Factors should explain at least 50 % of the variance
	?	Explained variance not mentioned
	−	Factors explain <50 % of the variance
Structural validity (IRT methods applied)	+	Residual correlations among the items after controlling for the dominant factor <0.20 OR Q3’s <0.37, item scalability >0.30, IRT model fit: G^2^ >0.01, no DIF for important subject characteristics (such as age, gender, education): McFadden’s *R* ^2^ <0.02, OR no non-uniform DIF
	?	Important statistics not reported
	−	Residual correlations among the items after controlling for the dominant factor ≥0.20 OR Q3’s ≥0.37, item scalability ≤0.30, IRT model fit: G^2^ ≤0.01, important DIF for important subject characteristics (such as age, gender, education): McFadden’s *R* ^2^ ≥0.02, OR non-uniform DIF
Hypothesis testing (convergent/divergent validity)	+	Correlations with instruments measuring the same construct ≥0.50 OR at least 75 % of the results are in accordance with the hypotheses AND correlation with related constructs is higher than with unrelated constructs
	?	Solely correlations determined with unrelated constructs
	−	Correlations with instruments measuring the same construct <0.50 OR <75 % of the results are in accordance with the hypotheses OR correlation with related constructs is lower than with unrelated constructs
Hypothesis testing (discriminative validity)	+	Differences in scores on the measurement instrument for all evaluated patient subgroups are statistically significant OR ≥75 % of results in accordance with hypotheses
	?	Some differences statistically significant, others not
	−	Differences in scores on the measurement instrument for all evaluated patient subgroups are not statistically significant OR <75 % of results in accordance with hypotheses
Cross-cultural validity	+	No differences in factor structure OR no important DIF between language versions
	?	Multiple group factor analysis not applied AND DIF not assessed
	−	Differences in factor structure OR important DIF between language versions
Responsiveness		
Responsiveness	+	Correlation with changes on instruments measuring the same construct ≥0.50 OR at least 75 % of the results are in accordance with the hypotheses OR AUC ≥0.70 AND correlations with changes in related constructs are higher than with unrelated constructs
	?	Solely correlations determined with unrelated constructs
	−	Correlations with changes on instruments measuring the same construct <0.50 OR <75 % of the results are in accordance with the hypotheses OR AUC <0.70 OR correlations with changes in related constructs are lower than with unrelated constructs

*MIC* minimal important change, *SDC* smallest detectable change, *LoA* limits of agreement, *ICC* intraclass correlation coefficient, *AUC* area under the curve, + positive rating, ? indeterminate rating, − negative rating

### Best evidence synthesis

If an instrument has been evaluated in multiple studies, findings will be synthesized if the characteristics of the included studies are sufficiently similar, if the results of the studies do not show significantly different or conflicting findings, and if the methodological quality of the included studies is sufficient [24]. The criteria for best evidence synthesis are outlined in Table 4.

**Table 4:** Levels of evidence for the overall adequacy of a measurement property adapted from [28]

Level	Rating	Criteria
Strong	+++, ? (strong) or −−−	Consistent findings in multiple studies of good methodological quality OR in one study of excellent methodological quality
Moderate	++, ? (moderate) or −−	Consistent findings in multiple studies of fair methodological quality OR in one study of good methodological quality
Limited	+, ? (limited) or −	One study of fair methodological quality
Conflicting	+/−	Conflicting findings
Unknown	?	Only studies of poor methodological quality

+ positive rating, ? indeterminate rating, − negative rating

### Generating recommendations for the use of QoL measurement instruments for eczema

For each instrument identified in the review, a standardized recommendation for usage or required future validation work will be made depending on the methodological quality of included studies and on the adequacy of the instrument (Table 5). According to the results of the HOME II meeting [9], all three criteria of the OMERACT filter [10], that is, truth, discrimination, and feasibility, have to be met by an outcome measure to be recommended by the HOME initiative. Although convergent/divergent and discriminative validity will be regarded separately throughout the review, the findings for these two aspects of hypothesis testing will be synthesized according to the following criteria: in case of conflicting ratings, the worse rating determines the overall rating for hypothesis testing; if one of the aspects obtains an indeterminate rating, the rating for the other aspect determines the overall rating for hypothesis testing.

**Table 5:** Adequacy criteria required for recommendation of QoL measures for eczema

Adequacy item (name)	Inclusion in OMERACT filter	Required rating for recommendation
Content validity	Truth	+
Structural validity	Truth	+
Hypotheses testing	Truth	+
Cross-cultural validity	Truth	+
Internal consistency	Discrimination	+
Reliability	Discrimination	+
Measurement error	Discrimination	+
Responsiveness	Discrimination	+

Four categories of recommendation will be made:

A. QoL measurement instrument meets all requirements and is recommended for use.
B. QoL measure meets two or more adequacy items, but performance in all other required adequacy items is unclear, so that the outcome measure has the potential to be recommended in the future depending on the results of further validation studies.
C. QoL measure has low adequacy in at least one required adequacy criterion (≥1 rating of “minus”) and therefore is not recommended to be used anymore.
D. QoL measure has (almost) not been validated. Its performance in all or most relevant adequacy items is unclear so that it is not recommended to be used until further validation studies clarify its adequacy.

Finally, we aim to identify one best (currently available) instrument to assess QoL in infants, one best (currently available) instrument to assess QoL in children, and one best (currently available) instrument to assess QoL in adolescents with eczema.

### Differences between this review and previously suggested methodology

We refined our eligibility criteria and made clear that generic QoL instruments will not be eligible for this review [12]. As this review will focus on infants, children, and adolescents, proxy-reported instruments will also be included.

Because interpretability and feasibility of a QoL instrument are very important for researchers and clinicians, we emphasized that corresponding information will be collected where presented. We also decided to regard different language versions of the same QoL instrument separately; this approach was also used in our previous review on adult QoL instruments [13] but initially not specified in the pertaining protocol. Content comparison of the included instruments will be done on content domain level instead of item level because a comparison table on item level would become unclear and confusing due to the multitude of data shown. Moreover, we decided to use the term “adequacy of the measurement properties” instead of “quality of the measurement properties.” For studies applying IRT methods, only internal consistency, construct validity, structural validity, and cross-cultural validity will be assessed, where applicable.

Important changes concern the adequacy criteria outlined in Table 3:

- For internal consistency, the indeterminate rating (“?”) was changed from “Dimensionality not known OR Cronbach’s alpha not determined” to “Cronbach’s alpha not determined” in order to avoid an overlap between the adequacy criteria and the COSMIN criteria for methodological quality. Adequacy criteria for studies using IRT methods were added.
- The IRT criteria for structural validity were enhanced with criteria on differential item functioning (DIF) [25]. If a study shows that there is no non-uniform DIF, this can now also result in a positive rating. Non-uniform DIF will be rated negatively according to the new criteria.
- Hypothesis testing was split into its two aspects convergent/divergent and discriminative validity, with separate criteria for each aspect, resulting in an overall rating for hypothesis testing in the end.
- The criteria developed by Terwee et al. for hypothesis testing will only be applied to convergent and divergent validity. For discriminative validity, another aspect of hypothesis testing, self-developed criteria were added. As the COSMIN initiative does not consider interpretability to be a formal measurement property, the adequacy criteria for interpretability were omitted [18].

An indeterminate rating for strong, moderate, and limited levels of evidence was added to the best evidence synthesis ratings each. This was done for scenarios where a QoL instrument would obtain an indeterminate rating for a certain measurement property. An indeterminate rating will be assigned to a measurement property if there is no clear evidence for either a positive or negative rating.

## Discussion

The proposed systematic review will yield a comprehensive assessment of measurement properties of existing QoL instruments in infants, children, and adolescents with eczema. We aim to arrive at a recommendation of one best instrument for infants, one best instrument for children, and one best instrument for adolescents, respectively. Rigorous and appropriate methods are vital to obtain meaningful, scientifically acknowledged results that form the basis to put forward such recommendations [6]. With good reason, researchers and clinicians demand that the development of a COS for eczema must adhere to high standards. We have made various efforts to satisfy these expectations. Firstly, the processes underlying this systematic review are transparent and systematic. Secondly, the involvement of at least two reviewers at each stage will assure quality of and reduce variability in the assessments. Another strength of the proposed research is the use of well-established methods and criteria, such as the COSMIN checklist, that have been successfully applied in a considerable number of previous systematic reviews. Furthermore, the international coverage of the contributing reviewers will increase the credibility of any findings.

In addition to the results obtained by best evidence synthesis, the feasibility of a questionnaire, e.g., number of items and time needed for administration, is another essential requirement for recommendation. This is also reflected by the fact that all three criteria of the OMERACT filter, i.e., truth, discrimination, and feasibility, need to be met by an outcome measure to be recommended by the HOME initiative [9, 10]. Truth and discrimination are reflected by the results from best evidence synthesis. Although there are no adequacy criteria for feasibility, information on feasibility will be collected throughout the review process and will be considered for the conclusions of our systematic review. Sufficient feasibility of a questionnaire is important for its inclusion in the proposed COS and the widespread implementation in future eczema trials.

Moreover, we may consider the popularity of a QoL instrument as an additional parameter for recommendation if several instruments are placed in category A and a decision to recommend one of them based solely on best evidence synthesis is not possible. A potential benefit of well-known and frequently applied QoL instruments could be that more data on the questionnaire’s feasibility and interpretability of its scores may be available compared to less popular instruments.

Whether or not we will be able to reach the goal of recommending one best instrument for each age group is unclear. It may well be that several instruments will meet the OMERACT filter criteria. If instruments lack important requirements, for instance, in relation to responsiveness or measurement error, they will not comply with the OMERACT filter criteria, and additional validation studies will need to be carried out before these instruments can be included in the COS. As a result, it could happen that our systematic review will only be able to identify priorities for further validation work instead of putting forward a clear recommendation for a certain QoL measurement instrument. Nonetheless, the findings of this systematic review will inform a consensus-finding process at the fifth meeting of the HOME initiative (HOME V) that will take place in São Paulo, Brazil, in 2017. Based on the findings of this work, we hope to be able to inform group discussion and consensus voting with the ultimate goal to endorse one instrument for each age group to be included in the core set of outcome measurement instruments for eczema.

## Additional files

Additional file 1:
**PRISMA-P 2015 checklist.** The completed PRISMA-P checklist for this protocol. Additional file 2:
**Search strings.** The search strings for MEDLINE (via PubMed) and EMBASE.

## Abbreviations

COS: core outcome set
COSMIN: COnsensus-based Standards for the selection of health Measurement INstruments
DIF: differential item functioning
HOME: Harmonising Outcome Measures for Eczema
HrQoL: health-related quality of life
IRT: item response theory
OMERACT: Outcome Measures in Rheumatology
PRISMA-P: Preferred Reporting Items for Systematic Reviews and Meta-Analyses Protocols
PRO: patient-reported outcome
PROSPERO: International Prospective Register of Systematic Reviews
QoL: quality of life
