# Supplementary material for: Measurement properties of quality of life measurement instruments for infants, children and adolescents with eczema: protocol for a systematic review
Source: Syst Rev. 2016 Feb 9;5:25. doi: 10.1186/s13643-016-0202-z (PMC4748496; doi:10.1186/s13643-016-0202-z)
Supplement: Additional file 2: — Search strings. The search strings for MEDLINE (via PubMed) and EMBASE. [file 13643_2016_202_MOESM2_ESM.doc]

Search string **Medline** (via PubMed)

**#1** (modified precision search terms by Terwee et al. 2009)

(instrumentation[sh] OR Validation Studies[pt] OR “reproducibility of results”[MeSH Terms] OR reproducib*[tiab] OR “psychometrics”[MeSH] OR psychometr*[tiab] OR clinimetr*[tiab] OR clinometr*[tiab] OR “observer variation”[MeSH] OR observer variation[tiab] OR “discriminant analysis” [MeSH] OR reliab*[tiab] OR valid*[tiab] OR coefficient[tiab] OR “internal consistency”[tiab] OR (cronbach*[tiab] AND (alpha[tiab] OR alphas[tiab])) OR “item correlation”[tiab] OR “item correlations”[tiab] OR “item selection”[tiab] OR “item selections”[tiab] OR “item reduction”[tiab] OR “item reductions”[tiab] OR agreement[tw] OR precision[tw] OR imprecision[tw] OR “precise values”[tw] OR test-retest[tiab] OR (test[tiab] AND retest[tiab]) OR (reliab*[tiab] AND (test[tiab] OR retest[tiab])) OR stability[tiab] OR interrater[tiab] OR inter-rater[tiab] OR intrarater[tiab] OR intra-rater[tiab] OR intertester[tiab] OR inter-tester[tiab] OR intratester[tiab] OR intra-tester[tiab] OR interobserver[tiab] OR inter-observer[tiab] OR intraobserver[tiab] OR intra-observer[tiab] OR intertechnician[tiab] OR intertechnician[tiab] OR intratechnician[tiab] OR intra-technician[tiab] OR interexaminer[tiab] OR inter-examiner[tiab] OR intraexaminer[tiab]) OR (intra-examiner[tiab] OR interassay[tiab] OR inter-assay[tiab] OR intraassay[tiab] OR intra-assay[tiab] OR interindividual[tiab] OR inter-individual[tiab] OR intraindividual[tiab] OR intra-individual[tiab] OR interparticipant[tiab] OR inter-participant[tiab] OR intraparticipant[tiab] OR intra-participant[tiab] OR kappa[tiab] OR kappa’s[tiab] OR kappas[tiab] OR “coefficient of variation”[tiab] OR repeatab*[tw] OR ((replicab*[tw] OR repeated[tw]) AND (measure[tw] OR measures[tw] OR findings[tw] OR result[tw] OR results[tw] OR test[tw] OR tests[tw])) OR generaliza*[tiab] OR generalisa*[tiab] OR concordance[tiab] OR (intraclass[tiab] AND correlation*[tiab]) OR discriminative[tiab] OR “known group” [tiab] OR “factor analysis”[tiab] OR “factor analyses”[tiab] OR “factor structure”[tiab] OR “factor structures”[tiab] OR dimensionality[tiab] OR subscale*[tiab] OR “multitrait scaling analysis”[tiab] OR “multitrait scaling analyses”[tiab] OR “item discriminant”[tiab] OR “interscale correlation”[tiab] OR “interscale correlations”[tiab]) OR ((error[tiab] OR errors[tiab]) AND (measure*[tiab] OR correlat*[tiab] OR evaluat*[tiab] OR accuracy[tiab] OR accurate[tiab] OR precision[tiab] OR mean[tiab])) OR “individual variability”[tiab] OR “interval variability”[tiab] OR “rate variability”[tiab] OR “variability analysis”[tiab] OR (uncertainty[tiab] AND (measurement[tiab] OR measuring[tiab])) OR “standard error of measurement”[tiab] OR sensitiv*[tiab] OR responsive*[tiab] OR (limit[tiab] AND detection[tiab]) OR “minimal detectable concentration”[tiab] OR interpretab*[tiab] OR (small*[tiab] AND (real[tiab] OR detectable[tiab]) AND (change[tiab] OR difference[tiab])) OR “meaningful change”[tiab] OR “minimal important change”[tiab] OR “minimal important difference”[tiab]) OR (“minimally important change”[tiab] OR “minimally important difference”[tiab] OR “minimal detectable change”[tiab] OR “minimal detectable difference”[tiab] OR “minimally detectable change”[tiab] OR “minimally detectable difference”[tiab] OR “minimal real change”[tiab] OR “minimal real difference”[tiab] OR “minimally real change”[tiab] OR “minimally real difference”[tiab] OR “ceiling effect” [tiab] OR “floor effect”[tiab] OR “Item response model”[tiab] OR IRT[tiab] OR Rasch[tiab] OR “Differential item functioning”[tiab] OR DIF[tiab] OR “computer adaptive testing”[tiab] OR “item bank”[tiab] OR “cross-cultural equivalence”[tiab] OR accepta*[tiab] OR “ease of use”[tiab] OR practica*[tiab] OR feasib*[tiab])

**#2**

(“addresses”[Publication Type] OR “biography”[Publication Type] OR “case reports”[Publication Type] OR “comment”[Publication Type] OR “directory”[Publication Type] OR “editorial”[Publication Type] OR “festschrift”[Publication Type] OR “interview”[Publication Type] OR “lectures”[Publication Type] OR “legal cases”[Publication Type] OR “legislation”[Publication Type] OR “letter”[Publication Type] OR “news”[Publication Type] OR “newspaper article”[Publication Type] OR “patient education handout”[Publication Type] OR “popular works”[Publication Type] OR “congresses”[Publication Type] OR “consensus development conference”[Publication Type] OR “consensus development conference, nih”[Publication Type] OR “practice guideline”[Publication Type]) NOT (“animals”[MeSH Terms] NOT “humans”[MeSH Terms])

**#3: #1 NOT #2**

**#4**

(quality of life[MH] OR quality of life[TW] OR health status[MH] OR health status[TW] OR "activities of daily living"[MH] OR activities of daily living[TW] OR life quality* OR daily life[TW]OR health level[TW] OR level of health[TW] OR patient reported outcome[TW] OR CDLQI[TW] OR IDQOL[TW])

**#5**

("dermatitis, atopic"[MeSH] OR atopic dermatitis[tiab] OR atopic eczema[tiab] OR eczema[MeSH] OR eczema[tiab] OR "neurodermatitis"[MeSH] OR Neurodermatitis[tiab]

OR skin diseases[MH] OR skin disease*[tiab] OR dermatology[tiab])

**#3 AND #4 AND #5**

Search string **Embase**

**#1**

exp instrumentation/ or exp validation study/ or exp reproducibility/ or reproducib$.mp. or exp psychometry/ or psychometr$.mp. or clinimetr*.mp. or clinometr$.mp. or exp observer variation/ or observer variation.mp. or exp discriminant analysis/ or exp reliability/ or reliab$.mp. or exp Validity/ or valid$.mp. or coefficient.mp. or internal consistency.mp. or (cronbach$ and (alpha or alphas)).mp. or item correlation.mp. or item correlations.mp. or item selection.mp. or item selections.mp. or item reduction.mp. or item reductions.mp. or agreement.mp. or precision.mp. or imprecision.mp. or precise values.mp. or (test-retest or (test and retest) or (reliab$ and (test or retest)) or stability or interrater or inter-rater or intrarater or intra-rater or intertester or inter-tester or intratester or intra-tester or interobserver or inter-observer or intraobserver or intra-observer or intertechnician or intertechnician or intratechnician or intra-technician or interexaminer or inter-examiner or intraexaminer or intra-examiner or interassay or inter-assay or intraassay or intra-assay or interindividual or inter-individual or intraindividual or intra-individual or interparticipant or inter-participant or intraparticipant or intra-participant or kappa or kappa$ or coefficient of variation or repeatab$ or ((replicap$ or repeated) and (measure or measures or findings or result or results or test or tests))).mp. or (generaliza$ or generalisa$ or concordance or (intraclass and correlation$) or discriminative or known group or factor analysis or factor analyses or factor structure or factor structures or dimensionality or subscale$ or multitrait scaling analysis or multitrait scaling analyses or item discriminant or interscale correlation or interscale correlations or ((error or errors) and (measure$ or correlat$ or evaluat$ or accuracy or accurate or precision or mean)) or individual variability or interval variability or rate variability or variability analysis or (uncertainty and (measurement or measuring)) or standard error of measurement or sensitiv$ or responsive$ or (limit and detection) or minimal detectable concentration or interpretab$ or (small$ and (real or detectable) and (change or difference)) or meaningful change or minimal important change or minimal important difference or minimally important change or minimally important difference or minimal detectable change or minimal detectable difference or minimally detectable change or minimally detectable difference or minimal real change or minimal real difference or minimally real change or minimally real difference).mp. or (ceiling effect or floor effect or Item response model or IRT or Rasch or Differential item functioning or DIF or computer adaptive testing or item bank or cross-cultural equivalence or practica$ or feasib$).mp.

#2 (Conference Abstract or Conference Paper or Conference Review or Editorial or Erratum or Letter or Note).pt.

**#3: #1 NOT #2**

**#4**

quality of life/ or quality of life.mp. or health status/ or health status.mp. or daily life activity/ or activities of daily living.mp. or life quality$.mp. or daily life.mp. or health level.mp. or level of health.mp. or health status/ or patient reported outcome.mp. or CDLQI.mp. or IDQOL.mp.

**#5**

exp Atopic Dermatitis/ or dermatitis, atopic.mp. or atopic dermatitis.mp. or exp eczema/ or atopic eczema.mp. or eczema, atopic.mp. or exp NEURODERMATITIS/ or neurodermatitis.mp. or skin disease/ or skin disease.mp. or dermatology/ or dermatology.mp.

**#3 AND #4 AND #5**
